# Pericytes Across the Lifetime in the Central Nervous System

**DOI:** 10.3389/fncel.2021.627291

**Published:** 2021-03-12

**Authors:** Hannah C. Bennett, Yongsoo Kim

**Affiliations:** Department of Neural and Behavioral Sciences, Penn State University, Hershey, PA, United States

**Keywords:** pericyte, brain, development, aging, blood brain barrier, regional heterogeneity, mouse

## Abstract

The pericyte is a perivascular cell type that encapsulates the microvasculature of the brain and spinal cord. Pericytes play a crucial role in the development and maintenance of the blood-brain barrier (BBB) and have a multitude of important functions in the brain. Recent evidence indicates that pericyte impairment has been implicated in neurovascular pathology associated with various human diseases such as diabetes mellitus, Alzheimer’s disease (AD), and stroke. Although the pericyte is essential for normal brain function, knowledge about its developmental trajectory and anatomical distribution is limited. This review article summarizes the scientific community’s current understanding of pericytes’ regional heterogeneity in the brain and their changes during major life stages. More specifically, this review article focuses on pericyte differentiation and migration during brain development, regional population differences in the adult brain, and changes during normal and pathological aging. Most of what is known about pericytes come from studies of the cerebral cortex and hippocampus. Therefore, we highlight the need to expand our understanding of pericyte distribution and function in the whole brain to better delineate this cell type’s role in the normal brain and pathological conditions.

## Introduction

Pericytes are a mural cell type that are highly abundant in the microvasculature in the central nervous system (CNS). These cells are tightly associated with the brain’s blood vessels and form one of the major components of the blood-brain barrier (BBB). The BBB, which forms during the embryonic and early postnatal period of development in mice, is comprised of endothelial cells, pericytes, and their shared basement membrane, as well as astrocytic endfeet (Ballabh et al., 2004). In humans, the development of the BBB corresponds to preterm infant development, or up to 32 weeks’ gestation (Semple et al., 2013). The BBB is crucial for regulating substances transported between the vessel lumen and the brain parenchyma and provides the nutrients, water, and oxygen required for proper brain function (Ballabh et al., 2004). Pericytes, in particular, form lock and socket junctions with endothelial cells and contribute significantly to the maintenance of the BBB (Gökçinar-Yagci et al., 2015). Various markers have been used to label pericytes, such as platelet-derived growth factor receptor β (PDGFRβ), neural glial antigen 2 (NG2), desmin, and aminopeptidase N (CD13; Birbrair, 2019; Brown et al., 2019). However, these markers are also expressed in other cell types, posing challenges in clearly labeling pericytes (Bondjers et al., 2006; Attwell et al., 2016). Moreover, pericytes have additional functions pertaining to toxin clearance, cytokine and chemokine production, as well as capillary blood flow regulation (Bell et al., 2010; Kamouchi et al., 2011; Gökçinar-Yagci et al., 2015; Sweeney et al., 2016; Trost et al., 2016; Brown et al., 2019). Several excellent reviews have recently summarized current evidence relating to the importance of pericytes, as well as their identification and versatile functions (Sims, 2000; Bergers and Song, 2005; Bell et al., 2010; Kamouchi et al., 2011; Gökçinar-Yagci et al., 2015; Attwell et al., 2016; Trost et al., 2016; Yamazaki and Mukouyama, 2018; Brown et al., 2019; Coelho-Santos and Shih, 2020). By analyzing the topics of embryonic and postnatal pericyte development, differences in brain regional vulnerability, as well as BBB and pericyte changes in normal and pathological aging, this review seeks to not only provide an integrated view of current knowledge but also emphasizes the need to further study pericyte populations across multiple brain regions to better delineate their roles in human disease.

## Embryonic Pericyte Development and Migration

**Figure 1:**
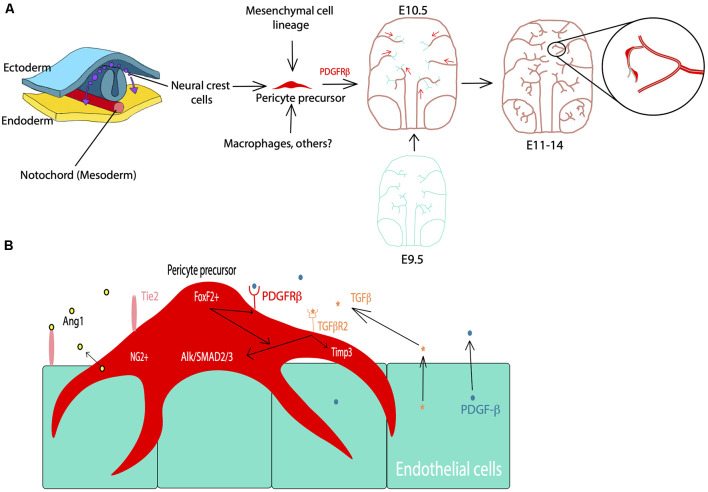
Pericyte development during the embryonic period. **(A)** Cell lineage of pericytes including neural crest cells, mesenchymal cells, and macrophages. PDGFRβ signaling facilitates pericyte migration during embryonic development starting at E10.5 in mice. Cyan: endothelial cells, Red: pericytes. **(B)** Examples of the major players in pericyte-endothelial interactions during embryonic development such as PDGFRβ signaling for pericyte development and migration, TGFβ signaling, Alk/SMAD2/3 and Timp3 signaling for pericyte-endothelial junctions and basement membrane formation, and FoxF2 for mediating PDGFRβ and TGFβ signaling during development.

BBB development, particularly that involves pericyte differentiation and migration, begins embryonically for both humans and rodents (Armulik et al., 2005). Most of what is known regarding CNS pericyte development is gleaned primarily from rodent studies in the brain and the retina. Although spinal cord pericyte development is also crucial for neurovascular development, potential differences between brain and spinal cord pericytes are largely understudied and both populations are presumed to be similar (Bartanusz et al., 2011; Picoli et al., 2019). CNS pericytes are thought to be primarily derived from the neural crest and mesenchymal cell lineages (Prazeres et al., 2018). However, a subset of the embryonic CNS pericyte population may arise from other sources, such as blood-borne macrophages (Yamamoto et al., 2017). At approximately embryonic day 10 (E10) of mouse development, there are hematopoietic lineage cells (CD31^+^F4/80^+^) containing well-known macrophage markers in the avascular region near the developing midbrain (Yamamoto et al., 2017). This particular subset of macrophages was tracked to the subventricular vascular plexus, within which they differentiated into cells expressing classic pericyte markers, including NG2, PDGFRβ, and desmin, and wrapped around the growing microvasculature (Yamamoto et al., 2017). Thus, the lineage of pericytes in the brain, and the CNS in general, may be heterogeneous (Figure 1A). Although the source of this cell type may vary, several studies have investigated the coordinated and essential roles of pericytes in the development of the neurovascular system (Armulik et al., 2005, 2011; Bergers and Song, 2005; Nakagomi et al., 2015).

Vascularization of the mouse brain is thought to begin at approximately E9.5 based on studies of the hindbrain, forebrain, and retina (Armulik et al., 2005, 2011; Paredes et al., 2018). In the spinal cord, vascularization begins slightly earlier, and current understanding of this process as well as differences between the BBB and blood spinal cord barrier have recently been reviewed (Bartanusz et al., 2011; Paredes et al., 2018; Picoli et al., 2019). Endothelial growth is largely driven by vascular endothelial growth factor (VEGF) signaling with the majority of mural cell precursors arising from the neural crest at around E10.5 (Armulik et al., 2005; Paredes et al., 2018). PDGFRβ is a receptor for the primary signaling growth factor for pericytes and is crucial for the migration and survival of these cells (Sweeney et al., 2016). PDGFRβ can be activated by both PDGF-B and PDGF-D isoforms, for which the ligand PDGF-B is primarily produced by endothelial cells (Sweeney et al., 2016). From E11.5 through E14, PDGF-B signaling plays an important role at all levels of the developing vasculature to help drive pericyte migration (Hellström et al., 2001). By E18.5 this expression is limited to capillaries, which then become a major site of pericyte cell density (Hellström et al., 2001). Also, there are several other pathways associated with the migration of these cells. For example, endothelial-derived transforming growth factor β (TGFβ) signaling and downstream components, such as Alk5/SMAD2/3, have been shown to play a role in the differentiation of pericytes, as well as the formation of the classic peg and socket junctions between pericytes and endothelial cells (Dave et al., 2018). The associated Alk5/Timp3 pathway in pericytes contributes to endothelial morphogenesis and regulation of basement membrane formation during embryonic development (Dave et al., 2018). TGFβ is also known to drive the incorporation of vascular smooth muscle cells (vSMCs) into larger arteries and venules (Allinson et al., 2012). Also, angiopoietin 1-Tie2 signaling mediates intercommunication between developing endothelium and pericytes (Teichert et al., 2017). More recently, precursor neural crest cells expressing PDGFRβ, described above, have also been shown to express forkhead box F2 (FoxF2; Reyahi et al., 2015). These cells are thought to comprise the precursors of mural cell types, including both pericytes and vSMCs (Armulik et al., 2005). FoxF2 is a factor that drives the development of mesenchymal cells of the gut, but it is also expressed by neural crest cells in brain development (Reyahi et al., 2015). The loss of FoxF2 perturbs the vascular system and BBB development by reducing TGFβ/Smad2/3 signaling as well as PDGFRβ expression (Reyahi et al., 2015). This indicates that FoxF2 plays a major role in the regulation of pericyte development as it has an impact on pathways essential for pericyte growth, differentiation, and survival. Additionally, molecules such as Gpr124, Zo-1, Notch, and others have also been implicated in pericyte and vascular development (Armulik et al., 2011; Wang et al., 2014; Yamazaki and Mukouyama, 2018; Zaitoun et al., 2019). Many of these signaling pathways associated with pericyte development are summarized in Figure 1B. Despite such a short developmental period, the BBB becomes functional by age E15.5 in mice and E17 in rats due to highly regulated pericyte-endothelial interactions (Daneman et al., 2010; Ben-Zvi et al., 2014). Although this structure is considered to be functional, its development is not quite finished at birth (Obermeier et al., 2013). In essence, pericytes play crucial roles in early vascular development, particularly by influencing the formation of the BBB and endothelial growth which are essential for proper neurovascular development.

## Postnatal Pericyte Development

**Figure 2:**
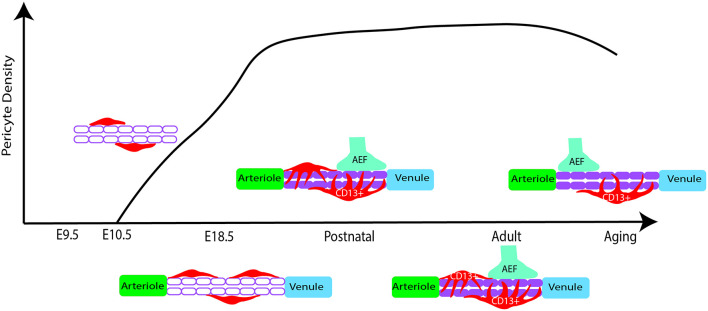
Pericyte density changes from early embryonic development to aging in mice. At E10.5 pericyte precursors are present and begin to migrate and develop along the developing vasculature. In late embryonic development, there is increased proliferation of pericytes, showing a higher density of cells in the capillaries. During early postnatal development, proliferation subsides while increased pericyte coverage of capillaries contributes to vessel stability. Pericytes also appear more similar to adult morphology. Astrocytic end feet (AEF) properly attach and contribute to brain barrier (BBB) maturation during this period. In adulthood, the population density is rather stable while there are differences by brain region. Finally, possible pericyte loss during aging. Red: pericyte, Purple: microvessel.

Following embryonic developmental migration and formation of the BBB, the microvasculature begins to mature in the early postnatal period in rodents (Figure 2), corresponding to the late gestational period in humans. After birth, endothelial cells show sharp proliferation to expand the vascular network which peaks at postnatal day 10 (P10) and drops by P25 (Harb et al., 2013). This is particularly apparent in the gray matter where the vascular density doubles by P20 and has a much higher pericyte density than the white matter (Zeller et al., 1996). Similarly, vascular branching increases around P10 and appears more similar to the adult brain by P25 (Harb et al., 2013). Alternatively, pericyte proliferation begins to decline in the postnatal period, while pericyte coverage of the vasculature continues to expand (Harb et al., 2013). Although pericyte proliferation is not as pronounced, pericyte coverage and vessel stability continue to mature during this period (Harb et al., 2013). By P1 in the mouse brain, pericytes have transitioned to partial coverage resembling that of adult pericyte morphology (Daneman et al., 2010; Obermeier et al., 2013). Additionally, pericyte expression of certain factors is crucial for proper development during this period. For example, loss of the pericyte-derived RBPJ transcription factor results in increased BBB permeability, excess TGFβ activation, cavernous malformations, and impaired vascular integrity (Diéguez-Hurtado et al., 2019). Finally, pericytes themselves are not mature until the postnatal period, as they do not begin to express CD13 until around P6 (Jung et al., 2018).

The BBB undergoes further maturation in the early postnatal period through the further attachment of astrocytic endfeet, with regulation and maintenance provided by pericytes and endothelial cells (Ma et al., 2012). Many of the factors that initiate the formation of vascular development *in utero* continue to refine these processes during the early postnatal period. For example, pericyte-expressed Tie2 plays an instrumental role in retinal angiogenesis, as Tie2 deletion delays postnatal angiogenesis and confers a migratory pericyte phenotype (Teichert et al., 2017). Additionally, PDGF-B/PDGFRβ signaling continues to promote pericyte survival and coverage of the microvasculature (Lindahl et al., 1997; Nikolakopoulou et al., 2017). There is also evidence to suggest pericytes regulate and guide postnatal endothelial expansion. This is particularly evident in the restriction of VEGF-induced endothelial sprouting, through the pericyte expression of VEGF receptor 1 (Darden et al., 2019). When VEGFR1 is specifically knocked out in pericytes there is an enlargement of vessels and associated angiogenic defects (Darden et al., 2019). Moreover, pericytes further facilitate endothelial function and BBB integrity through regulation of a key BBB regulator called endothelial major facilitator superfamily domain-containing 2a (Mfsd2a) expression, which serves to suppress transcytosis in these cells (Ben-Zvi et al., 2014). Loss of this BBB-specific protein serves to perturb tight junctions and increase endothelial transcytosis (Ben-Zvi et al., 2014). The above is only a snapshot of this concerted effort to develop and mature the BBB but also provides context for the important role pericytes play in this process. However, there are still gaps in our knowledge of these vascular developmental processes, and how this relates to neurodevelopmental disorders. For example, what is an underlying mechanism to regulate pericyte density in the early postnatal period? Do pericytes undergo a pruning stage of postnatal development that is similar to endothelial cells and neurons? How might disturbed processes in vascular development relate to neurodevelopmental disorders? Although there is much more to investigate, this vascular growth trajectory is important for understanding how the brain develops and how this development progresses in parallel with neuronal development. For further reading, the development of cerebrovascular structure and the neurogliovascular unit during the postnatal period was recently summarized in several excellent reviews (Paredes et al., 2018; Coelho-Santos and Shih, 2020).

## Regional Differences of The Pericyte and Associated Vulnerability in The Adult Cns

Pericytes are present in most regions of the adult CNS and yet the distribution of this cell population and its associated characteristics are a major area of exploration. Although several studies have utilized *in vivo* 2-photon imaging and immunohistochemical methods to study these cells during development, aging, and disease pathology, very few studies have described pericyte population differences across brain regions of the adult mouse CNS, let alone in humans. However, pericyte populations are likely to be variable across different tissues and organs (Sims, 2000). Studies of wild-type mice demonstrate that pericyte density is decreased in the spinal cord compared to the brain, with regional variations between spinal cord levels (Winkler et al., 2012). Major challenges associated with investigating pericyte population variability, particularly in the brain, include a lack of specific cellular markers for pericytes, controversy in the field about pericyte classification, and technological limitations in quantitative cell type brain mapping (Sims, 2000; Attwell et al., 2016; Yamamoto et al., 2017). The use of transgenic PDGFRβ-Cre and NG2-Cre mouse lines has helped to demonstrate that pericytes are generally present throughout the whole mouse brain (Hartmann et al., 2015). Additionally, pericyte distribution differs by cortical layer within the cerebral cortex, indicating that these cells may not be uniformly located along the vasculature (Hartmann et al., 2015). The morphology of these cells can vary as well (Sims, 2000; Hartmann et al., 2015; Berthiaume et al., 2018). For example, pericytes within the cerebral cortex can accommodate several morphologies, including the classical *en passant* or helical pericyte, as well as mesh pericytes which are more difficult to visually distinguish from vSMCs (Hartmann et al., 2015). Moreover, pericyte subtypes with different morphologies cover different vascular territories (Grant et al., 2019). These different pericyte morphologies and the terms used to categorize them have been recently described (Berthiaume et al., 2018). The phenotypic and molecular differences between pericytes and other mural cells have been further explored thanks to advances in single-cell RNA sequencing (Vanlandewijck et al., 2018; Zeisel et al., 2018). There seems to be a continuum in the transitions between different types of mural cells across the vasculature, in which the transition from arteriolar vSMCs to pericytes can occur between neighboring cells (Vanlandewijck et al., 2018; Grant et al., 2019). Additionally, transcriptional differences exist between arteriolar- and venule-associated vSMCs, as well as pericytes. However, there appears to be a lack of transcriptional heterogeneity within the pericyte population itself (Vanlandewijck et al., 2018).

Of course, it is necessary to note that this uniformity also depends on the definition of the pericyte that is used, which remains intensely debated in the field (Attwell et al., 2016). For example, the inclusion of smooth muscle actin-expressing contractile PDGFRβ+ cells located within the arteriolar branches of the capillary bed as pericytes may expand the transcriptional diversity of this cell population (Hall et al., 2014; Attwell et al., 2016). Additionally, another transcriptomic study indicates that there are three pericyte subtypes with type 1 being the most numerous throughout the brain while type 3 pericytes are more similar to vascular smooth muscle cells, as per their respective expression profiles (Zeisel et al., 2018). To complicate things further, studies continue to demonstrate that pericytes, even capillary pericytes, may also have vasomotor functions to regulate cerebral blood flow and neurovascular coupling (Alarcon-Martinez et al., 2020; Nelson et al., 2020). Recently, a pericyte-specific mouse model was developed using a double promoter method to exclusively label pericytes and to highlight the catastrophic effects of pericyte ablation (Nikolakopoulou et al., 2019). This new animal model will help to further delineate the complicated categorization of pericytes in the future. Additional comprehensive transcriptomic and cell-type mapping studies can also help to develop a more well-rounded definition of the pericyte by uncovering specific characteristics of this intriguing and rather unique cell population.

**Figure 3:**
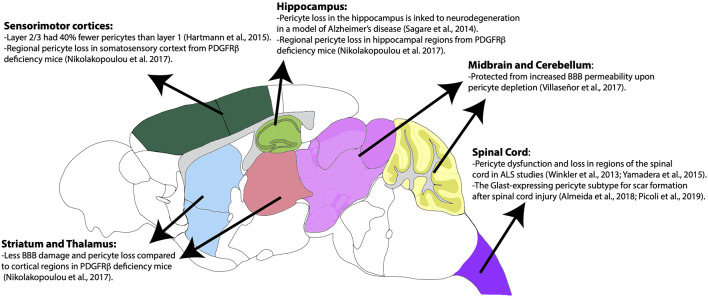
Regional heterogeneity of pericyte distribution and vulnerabilities. Brain regions are color-coded according to the Allen Mouse Brain Atlas. Brain regions with heterogenous distribution and vulnerability are highlighted.

Pericytes have heterogeneous vulnerability across regions of the CNS in pathological conditions as well. For example, a study using Pdgfrβ^F7/F7^ mice, a model of pericyte deficiency, showed that there are regional differences in pericyte loss, capillary length reductions, and BBB breakdown (Nikolakopoulou et al., 2017). Importantly, these losses occurred much earlier and more significantly in the somatosensory cortex and hippocampus, while the thalamus and striatum were less affected (Nikolakopoulou et al., 2017). Although vSMCs also express PDGFRβ, these populations appeared to be less affected at time points in which pericyte loss was already present (Nikolakopoulou et al., 2017). This could indicate that there is a difference in the vulnerability of cortical vs. subcortical pericyte populations. The use of a similar mouse model of pericyte deficiency showed that there is also BBB permeability heterogeneity by region upon pericyte loss (Villaseñor et al., 2017). In this study, there was increased BBB permeability, measured by both Evan’s blue dye and IgG leakage, in the areas of the cortex, striatum, and hippocampus, while areas such as the cerebellum and midbrain were less prominent (Villaseñor et al., 2017). Importantly, this difference was not due to differences in local pericyte coverage or changes in tight junction integrity (Villaseñor et al., 2017). The reasons behind this regional difference in permeability following significant pericyte loss should be further explored. Many of the above findings are summarized in Figure 3. Also, pericytes appear to be more susceptible to ischemic injury compared to endothelial cells in both the gray and white matter of rat neocortical and cerebellar brain slices, respectively (Hall et al., 2014). This susceptibility is not limited to the brain, as pericytes in the spinal cord are also quite heterogeneous and implicated in several pathologies (Bartanusz et al., 2011; Almeida et al., 2018; Picoli et al., 2019). In fact, in studies of Amyotrophic Lateral Sclerosis (ALS), there is evidence of pericyte dysfunction and loss in particular regions of the spinal cord such as the ventral horn (Winkler et al., 2013; Yamadera et al., 2015). Moreover, pericytes have been a focus of study in spinal cord injury due to their role as a potential therapeutic target and pericyte Glast-expressing subtype involvement in scar formation in response to injury (Almeida et al., 2018; Picoli et al., 2019). Differences in cell lineage and normal behaviors associated with these spinal cord pericyte subtypes would be invaluable to expanding the field’s understanding of this population. Summarization of this pericyte heterogeneity in the spinal cord is also shown in Figure 3. This CNS pericyte vulnerability and evidence of cell loss suggest that there are cell-specific and regional differences in terms of microvascular vulnerability to injury or disease. However, several questions about CNS pericyte populations remain. How do pericyte morphology, function, and regional vulnerability differ if the population seems to be transcriptionally uniform? Are there fewer pericytes in circumventricular organs, as would be expected given the known “leakiness” of these brain regions (Wilhelm et al., 2016)? How do spinal cord pericytes differ from brain pericytes in terms of their cell lineage? One thing is certain, further studies are required to better understand pericyte and mural cell populations in general.

## Bbb and Pericyte Changes in Normal Aging

Given the apparent evidence for pericyte vulnerability, it is possible that aging may influence the BBB and pericyte population changes. Aging itself is rather complex and further complicated by diseases that seem to be inextricably linked to the aging process in humans. Some of these pathologies include dementia, a vast array of cerebrovascular diseases, endocrine diseases, and autoimmune disorders. Not only does aging affect the body systemically, but it also has implications within the brain. Aging is known to have a multitude of effects on neuronal and glial cells, including DNA damage, morphological changes, activation of inflammatory cascades, and impairments in function, which have been recently reviewed (Knox, 1982; Peters et al., 1991; Palmer and Ousman, 2018; Valles et al., 2019). Studies also indicate that aging may contribute to changes in the neurovasculature and the BBB (Li et al., 2019). This is often linked to the consistent finding of chronic inflammation that develops during aging, typically referred to as immunoaging (Fjell et al., 2014; Palmer and Ousman, 2018; Valles et al., 2019). For example, in C57 mice aged 24 months, there is increased BBB permeability and elevated neurovascular inflammation in hippocampal and cortical regions when compared to young controls (Jansson et al., 2014; Duan et al., 2018). Furthermore, changes in inflammatory molecule expression during normal aging lead to loss of tight junction integrity and increased BBB leakiness (Jansson et al., 2014; Duan et al., 2018). The findings of this study support previous work in humans showing that healthy participants greater than 60 years of age had increased BBB permeability (Farrall and Wardlaw, 2009). Additionally, an MRI study found that even individuals without cognitive impairment had age-dependent decreases in BBB integrity in the hippocampus, particularly the dentate gyrus and CA1 region, while other cortical and subcortical regions were less affected (Montagne et al., 2015). Evidence of these age-related neurovascular changes in humans is particularly striking, especially when considering that this may be occurring in normal aging. Finally, a notable study showed that even aside from BBB leakiness, there is an age-related shift in transcytosis at the BBB, involving a decrease in receptor-mediated transport, which can heavily influence brain homeostasis (Yang et al., 2020). This study also suggested that this age-related change in transcytosis may be influenced by pericyte loss (Yang et al., 2020). Needless to say, the impacts of aging on the BBB are likely to be due to changes in its components.

There is also evidence to suggest that aging is also associated with changes in CNS pericyte populations themselves, as well as their functions and interactions with other cell types. Even in the absence of pathological states, aging in mice is associated with pericyte dysfunction (Peters et al., 1991; Hughes et al., 2006; Elahy et al., 2015). Various studies indicate that during aging, pericytes may have impaired interactions with endothelial cells (Armulik et al., 2005; Hughes et al., 2006; Elahy et al., 2015). Moreover, aging-related pericyte dysfunction has been associated with mitochondrial, migratory, and phenotypic changes (Armulik et al., 2011; Erdö et al., 2017; Jackson et al., 2017; Yamazaki and Mukouyama, 2018). For example, studies have noted ultrastructural changes in these cells, such as lipofuscin inclusions, changes in mitochondrial size, increased smooth muscle expression, and overall changes to pericyte structure and morphology (Knox, 1982; Bar, 1985; Peters et al., 1991; Hughes et al., 2006; Elahy et al., 2015). A more severe change in neurovascular function is pericyte loss, which has previously been suggested to occur in normal aging (Peters et al., 1991; Farrall and Wardlaw, 2009). Pericyte loss is well known to have detrimental effects on BBB permeability, which could also explain compromised neurovascular regulation demonstrated in previous aging studies (Bell and Zlokovic, 2009; Bell et al., 2010; Elahy et al., 2015). Additionally, pericyte loss impacts the microcirculation within the brain, leading to oxidative stress and hypoxia in these oxygen-starved areas (Bell et al., 2010). However, pericyte loss upon aging remains controversial, as pericyte populations appeared to be similar when compared in 2-month-old and 22-month-old mice, according to a recent transcriptomic study (Ximerakis et al., 2019). Of course, it is important to keep in mind that this study does not account for potential subtypes or regional differences in this cell population. Therefore, it will be necessary to resolve this controversy to better distinguish changes in normal aging from those that occur in pathological processes.

## Bbb and Pericyte Dysfunction in Pathological Aging

Pathological states, particularly neurodegenerative diseases, have increased damage to the cerebral microvasculature and pericytes. Notably, increased BBB permeability and inflammation have been demonstrated in human patients with Alzheimer’s disease (AD) and AD mouse models (Bell and Zlokovic, 2009; Halliday et al., 2016; Kisler et al., 2017; Brown et al., 2019). Given that these changes are also present in normal aging, it is likely that these pathological processes further compromise neurovascular function. For example, in the amyloid precursor protein (APP) overexpression mouse model of AD, pericyte loss accelerated Amyloid β (Aβ) pathology and neurodegeneration with deteriorated memory performance (Sagare et al., 2013). Several studies have further demonstrated that pericyte deficiency is associated with neurodegeneration in both the gray and white matter of the brain (Bell et al., 2010; Montagne et al., 2018; Nikolakopoulou et al., 2019). Moreover, pericytes express pleiotrophin which is a neurotrophic growth factor that is crucial for neuronal health (Nikolakopoulou et al., 2019). This suggests that pericytes’ roles may expand beyond BBB functions to directly contribute to neuronal health in the brain. Furthermore, this provides evidence that vascular and pericyte dysfunction occurs before neurons begin to show evidence of damage and cell loss. Additionally, Aβ oligomers have been shown to signal to pericytes, thereby causing capillary constriction (Nortley et al., 2019). This demonstrates a crucial link between the toxic accumulation of cellular byproducts and associated neurovascular dysfunction. Moreover, pericytes are known to clear Aβ through receptor-mediated endocytosis, involving the low-density receptor-related protein 1 (LRP1), which is an important protein in Aβ homeostasis at the BBB (Shibata et al., 2000; Ma et al., 2018). Clearly, pericytes are instrumental in the regulation of Aβ accumulation and may provide a key link in the pathophysiology of AD.

Importantly, apolipoprotein E4 (APOE4), which is a major susceptibility factor for the development of AD has also been linked to BBB dysfunction and injury to pericytes in both AD models and in human carriers (Lane-Donovan and Herz, 2017; Uddin et al., 2019; Montagne et al., 2020). For instance, APOE4 is linked to BBB breakdown through the activation of a proinflammatory cascade in pericytes through upregulation of the cyclophilin A-mediated nuclear factor κB-matrix metalloproteinase-9 pathway (Bell et al., 2012; Halliday et al., 2016). This not only helps to clarify the role of pericytes in neuroinflammatory states but could also relate to the inflammatory processes that are classically associated with AD pathology. Recently, APOE4 carriers were shown to have increased evidence of pericyte injury, measured by soluble PDGFRβ (sPDGFRβ), which was predictive of cognitive decline independent of Aβ and pTau status (Sagare et al., 2015; Sweeney et al., 2015; Nation et al., 2019; Montagne et al., 2020). Moreover, a new method for detecting sPDGFRβ in both the plasma and cerebrospinal fluid can be clinically useful for identifying pericyte injury in neurovascular diseases (Sweeney et al., 2020). These studies further support that pericytes have an important role in neurodegenerative disease and spark additional questions relating to the role of pericytes in AD. Furthermore, pericyte loss has long been associated with diabetic retinopathy and diabetes mellitus is known to be a major risk factor for the development of AD (Pfister et al., 2008, 2010; Hayden, 2019). It is not surprising to think that neurovascular dysfunction likely serves as a unifying factor in the development of these disease processes. It would be intriguing to know whether the sPDGFRβ marker for pericyte injury could also be identified in other diseases associated with neurovascular dysfunction, such as diabetes, and how this could serve clinically to potentially identify patients with higher risk of cognitive decline. However, pericytes are not only implicated in AD, as evidence relating to pericyte involvement in various human diseases has recently been described in several excellent reviews (Table 1).

**Table 1:** Pericyte involvement in human diseases.

System	Disease	Associated regions	References
Neurological	Epilepsy	Temporal lobe (most commonly)	Sweeney et al. (2018b)
	Spinal cord injury	The spinal cord, CNS	Bartanusz et al. (2011), Almeida et al. (2018), Sweeney et al. (2018b) and Picoli et al. (2019)
Neurodegenerative	Alzheimer’s disease	Hippocampus, entorhinal cortex, basal forebrain	Girouard and Iadecola (2006); Zlokovic (2008); Bell and Zlokovic (2009), Armulik et al. (2011), Winkler et al. (2011), Baloyannis and Baloyannis (2012), Rosenberg (2014), Di Marco et al. (2015), Lane-Donovan and Herz (2017), Newcombe et al. (2018), Sweeney et al. (2018a), Lendahl et al. (2019), Nation et al. (2019) and Montagne et al. (2020)
	Vascular dementia	Commonly vascular regions of the middle cerebral artery	Montagne et al. (2020), Moretti and Caruso (2020) and Uemura et al. (2020)
	Parkinson’s disease	Substantia nigra pars compacta	Erdö et al. (2017), Sweeney et al. (2018b) and Li et al. (2019)
	Traumatic brain injury/traumatic encephalopathy	Frontal lobe regions	Dore-Duffy et al. (2000) and Main et al. (2018)
Cardiovascular	Diabetes mellitus, DM Retinopathy	Retina	Pfister et al. (2008), Persidsky et al. (2016), Laredo et al. (2019) and Rhea and Banks (2019), Liu et al. (2020)
	Hypertension	The vascular territory of the middle cerebral artery	Knox (1982), Girouard and Iadecola (2006) and Hirunpattarasilp et al. (2019)
	Small vessel disease	Microvasculature	Hogan and Feeney (1963), Neurology Working Group of the Cohorts for Heart and Aging Research in Genomic Epidemiology (CHARGE) Consortium, the Stroke Genetics Network (SiGN) and the International Stroke Genetics Consortium (ISGC) (2016) and Moretti and Caruso (2020)
	Stroke, Ischemia	Several CNS regions	Girouard and Iadecola (2006), Lucke-Wold et al. (2014), Neurology Working Group of the Cohorts for Heart and Aging Research in Genomic Epidemiology (CHARGE) Consortium, the Stroke Genetics Network (SiGN) and the International Stroke Genetics Consortium (ISGC) (2016) and Hu et al. (2017)
Immunological	(ALS)	Spinal cord	Zlokovic (2008) and Winkler et al. (2011)
	Multiple sclerosis (MS)	White matter tracts of the brain	Ortiz et al. (2014)
	General neuroinflammation	Blood-brain barrier (BBB)	Jansson et al. (2014), Persidsky et al. (2016), Rustenhoven et al. (2017) and Newcombe et al. (2018)
CNS Infection	Viral: HIV, CMV	BBB	Nakagawa et al. (2012) and Bertrand et al. (2019)
	Bacterial infections	BBB	Guijarro-Muñoz et al. (2014)
Cancer of the CNS	Glioblastoma/Glioma	BBB, midline cortical regions	Liebner et al. (2018) and Valdor et al. (2019)

## Conclusion

The pericyte population of the brain is not static during our lifetime. These cells arise from heterogeneous sources and undergo significant cell proliferation and migration throughout embryonic development. Unlike other cell types, they have slowed proliferation in early postnatal stages, while also reaching their processes extensively across the microvasculature. Finally, in adulthood pericytes show varying morphologies and characteristics, and yet, this cell population is transcriptionally similar across the board. Moreover, mounting evidence continues to demonstrate the importance of these cells during the lifespan and in all regions of the mammalian brain. Pericytes are also becoming increasingly recognized for their potential roles in the pathophysiology of various human diseases, particularly those impacted by the aging process. With each new study, these mural cells become more intriguing and unique. To better understand this cell population and the brain vasculature as a whole, research cannot only be restricted to particular regions as this does not encompass the systemic impact of the brain vasculature.

## Author Contributions

HB and YK conceptualized the manuscript. HB wrote the initial manuscript draft and figures. YK handled the funding and critically revised the manuscript. All authors contributed to the article and approved the submitted version.

## Conflict of Interest

The authors declare that the research was conducted in the absence of any commercial or financial relationships that could be construed as a potential conflict of interest.

## Abbreviations

aminopeptidase N: CD13
CNS: central nervous system
BBB: blood-brain barrier
PDGFRβ: platelet-derived growth factor receptor β
PDGF-β: platelet-derived growth factor β
NG2: neural glial antigen 2
vSMC: vascular smooth muscle cell
VEGF: vascular endothelial growth factor
VEGFR1: vascular endothelial growth factor receptor 1
TGFβ: transforming growth factor β
FoxF2: forkhead box F2
Mfsd2a: endothelial major facilitator superfamily domain-containing 2a
postnatal day #: P#
embryonic day #: E#
AD: Alzheimer’s disease
APP: amyloid precursor protein
Amyloid β: Aβ
LRP1: low-density receptor-related protein 1
APOE4: apolipoprotein E4
sPDGFRβ: soluble PDGFRβ.
